# Varicella Outbreak in an Indian Couple Living in Germany Caused by VZV Clade VI Acquired during a Trip to The Netherlands

**DOI:** 10.1155/2012/838241

**Published:** 2012-03-14

**Authors:** Malgorzata Kolesnik, Bernd Bonnekoh, Ina Tammer, Harald Gollnick, Andreas Sauerbrei

**Affiliations:** ^1^Clinic and Policlinic for Dermatology und Venereology, Otto-von-Guericke University Magdeburg, 39120 Magdeburg, Germany; ^2^Institute of Medical Microbiology, Otto-von-Guericke University Magdeburg, 39120 Magdeburg, Germany; ^3^Institute of Virology and Antiviral Therapy, German Reference Laboratory for HSV and VZV, Jena University Hospital, Friedrich Schiller University Jena, 07745 Jena, Germany

## Abstract

Varicella-zoster virus (VZV), the cause of varicella and zoster, is divided into five major clades and four provisional clades, the latter of which have been rarely reported worldwide to date. We present a varicella outbreak by the provisional clade VI within an Indian couple in Germany returning from a trip to Amsterdam. To the best of our knowledge, this is the first case of varicella by the VZV clade VI described in Germany, but the disease was acquired in The Netherlands.

## 1. Introduction

Varicella-zoster virus (VZV), a member of the genus *Varicellovirus* within the subfamily *Alphaherpesvirinae* and the *Herpesviridae *family, causes two distinct diseases. Primary infection results in typical signs of varicella also termed as chickenpox, and zoster is caused by endogenous reactivation after the virus has established lifelong latency in trigeminal and dorsal root ganglia. As a function of geographic distribution, VZV has been separated into the five major clades 1–5 confirmed by full-genome sequencing. In addition, there have been proposed four novel clades VI to IX still to be confirmed by further complete sequences [1, 2]. These provisional clades have been rarely reported to date [2, 3]. The paper presented is the first description of varicella caused by the VZV clade VI in Germany. This and the striking clinical picture of relatively large bullous lesions of up to 8 mm in diameter seem to be worth to be presented as a case report.

## 2. Case Report

### 2.1. Patients' History

A married couple from India, a 32-year-old man and a 25-year-old woman, was admitted to the dermatologic clinic in July 2010 since both had developed varicella. The woman was pregnant at week 34 of gestation. In both patients, an immunodeficiency was not known. One week before admission, the patients had visited a friend who was also coming from India, but he was a permanent resident in Amsterdam, The Netherlands, at that time. Two days after the meeting, the index case had developed a vesicular exanthema, a typical clinical sign of varicella. However, information on the possible source of the virus (contact person) and the vesicular characteristics of the exanthema was not available.

### 2.2. Clinical Examination

Seven days after the contact with the index case, the Indian couple was admitted to the hospital. The man presented with diffuse exanthema consisting of vesicles with a narrow erythematous rim as well as a vesicular, erosive enanthema of the oral mucosa. As a striking finding, there were single large bullous lesions, measuring up to 8 mm in diameter. Additionally, the patient suffered from fever and adynamia. The woman showed multiple papulovesicular skin lesions. Overall, the male patient showed an initially severe course of varicella, whereas the female patient had a relatively mild course.

### 2.3. Laboratory Diagnostics

Routine blood testing revealed hepatopathy with elevated serum concentration of alanine aminotransferase (2.5x of reference) for the man. Testing of vesicle fluid for VZV DNA using polymerase chain reaction [4] was positive in both patients. Sera were tested for the presence of VZV-specific antibodies using enzyme-linked immunoabsorbent assays Enzygnost^®^ Anti-VZV/immunoglobulin (Ig) G and Anti-VZV/IgM (Siemens Healthcare Diagnostics, Eschborn, Germany) at an early point of time, 5 days after the first cutaneous lesions appeared. IgM und IgG were negative in the man, whereas the woman's IgM was positive and IgG was negative. Unfortunately, a second serum sample from the convalescence phase was not analyzed. The molecular-biological characterization of the virus using restriction fragment length polymorphism analysis of the open reading fames (ORF) 38 and 54 [5] as initial step revealed the wild-type VZV pattern  *PstI* ^+^ *Bg*l*I* ^−^. Further characterization of ORFs 1, 21, 22, 50, 54, and 60 by the scattered single nucleotide polymorphism (SNP) method [6] resulted in typical SNPs of the novel provisional VZV clade VI (Table 1) which was detected in both patients.

#### 2.3.1. Treatment and Course

The patients received acyclovir intravenously at a dosage of 5 mg per kg body weight 3 times daily for 7 days. The man was treated additionally with systemic antibiotics. Zinc sulphate gel was used as topical antiseptic therapy. Gynecological examination of the woman showed a regular pregnancy. Both patients recovered without complications. Subsequently, the female patient gave mature birth to a healthy child.

## 3. Discussion

Varicella is a common highly infectious disease with an average age of manifestation differing due to geographical location. In Europe and North America, varicella is seen in 90% of the cases before the age of 10 years [7]. However, in tropical and subtropical countries, such as in India, varicella is occurring mostly in adolescents and young adults [8]. VZV as the etiological agent of varicella represents one viral serotype, but it is divided into different genetic clades due to viral evolution. The genomes of the five main VZV clades characterized by the Arabic numerals 1–5 are completely sequenced, while genome sequencing data for the four provisional VZV clades assigned to the Roman numerals VI to IX are partially incomplete [1–3]. Presently, in Germany, the European clades 1 and 3 are prevailing in varicella with a frequency of 30% and 46%, respectively, followed by African/Indian clade 5 (21%) and clade 2 (0.9%) causing varicella by the vaccine Oka strain after varicella vaccination [9]. By contrast, VZV wild-type strains of clade 2 are typical for Japan and China [3].

Since the incubation period of varicella is up to 21 days, it is beyond doubt that the Indian couple described in this paper acquired varicella during their trip to The Netherlands. Both patients developed the disease within one week after VZV exposure to the index case. The novel provisional VZV clade VI was identified as the etiological agent of varicella. The provisional clade VI is considered to be a combination of the European clade 1 or 3 with the African/Indian clade 5 or clade 4 that has been found predominantly in countries of South and Central America [3, 10]. Till now, clade VI has been detected only in 11 cases in Europe, that is, two cases in France, two cases in Italy, and seven cases in Spain [10, 11]. In addition, there were four cases in Australia and one case in Mexico [12–14]. To our knowledge, the two patients of this paper are the first cases of VZV infection by clade VI described in Germany, but the virus was acquired during a trip to Amsterdam. Since the index case was a permanent citizen of The Netherlands at this time, it is not clear if the virus circulates in The Netherlands or the index case was infected by immigrants who imported the virus from abroad. Thus, the future epidemiology of VZV clades in varicella can be influenced continuously by travel activities and migration, but also by vaccination policies, with a presumptive subsequent influence upon herpes zoster as a secondary VZV manifestation.

Two years ago, we observed a varicella outbreak in Indian students in Magdeburg caused by the African/Indian VZV clade 5 [15]. Clinically, varicella in these students was also characterized by relative large bullous lesions up to 8 mm in diameter, similarly to the current male patient affected by VZV clade VI. This may give some reason to check future cases carefully to find out to what extent such relatively large bullous lesions could indeed be clinically indicative of infections caused by VZV clade 5 or VI. This is important in so far as the different major VZV clades have not been distinguished by any different virulence to date. However, since the woman appeared to display more ordinary lesions, the bullous lesions might also be related to host genetic or other factors such as differences in viral loads that were not analyzed. In conclusion, further clinical studies are required to verify any associations between VZV clades and the characteristics of cutaneous varicella lesions.

##  Consent 

The patients have given their informed consent for the paper published.

##  Authors' Contribution 

Malgorzata Kolesnik and Bernd Bonnekoh contributed equally to this paper.

## Figures and Tables

**Table 1:** Single-nucleotide polymorphisms in VZV strains of the novel provisional clade VI detected in two Indian patients with varicella in comparison to other VZV clades. ORF: open reading frame, ND: not done.

Clades/ Reference	ORF 1			ORF 21	ORF 22					ORF 50	ORF 54	ORF 60
strains	508	685	790	33725	37902	38019	38055	38081	38177	87841	95241	101464
1/Dumas	C	G	T	T	A	G	T	A	G	C	T	C
2/pOka	C	A	C	C	G	G	C	C	A	T	C	C
2/vOka	C	A	C	C	G	G	C	C	A	T	C	C
3/VZV11	C	G	T	C	A	G	T	A	G	T	T	C
4/VZV8	C	A	C	C	A	G	C	C	A	T	C	C
5/CA123	C	G	C	C	A	G	T	C	G	T	C	A
**VI**	**T **	**T **	**G**	**C**	**A**	**G**	**T **	**C**	**A**	**T**	**T**	**C**
VII	ND	ND	ND	ND	ND	G	T	C	G	ND	C	ND
VIII	C	A	C	C	A	G	T	C	A	T	T	C
IX	C	G	C	C	A	G	C	A	G	T	C	C
**Indian patients**	**T **	**T **	**G**	**C**	**A**	**G **	**T**	**C**	**A**	**T**	**T**	**C**
